# Ectropion bilatéral sévère compliquant une sclérodermie: à propos d'un cas

**DOI:** 10.11604/pamj.2014.18.125.4701

**Published:** 2014-06-09

**Authors:** Jaja Zineb, Daoudi Rajae

**Affiliations:** 1 Université Mohammed V Souissi, Service d'Ophtalmologie A de l'hôpital des spécialités, Centre Hospitalier Universitaire, Rabat, Maroc

**Keywords:** Ectropion, sclérodermie, sécheresse oculaire, ectropion, sclerodermie, ocular dryness

## Image en medicine

L'ectropion est une éversion du bord libre des paupières. L'ectropion peut être congénital ou acquis; le plus souvent il est d'origine sénile ou paralytique ou post traumatique. Nous présentons le cas d'une patiente suivie pour sclérodermie depuis son enfance et nous a été envoyée pour son ectropion bilatéral. L'examen clinique a retrouvé un aspect de peau cartonnée au niveau de son visage et tout son corps caractéristique de sa maladie et un ectropion bilateral avec larmoiment secondaire à la sécheresse oculaire. On voulait tenter une chirurgie plastique pour son ectropion mais la greffe de peau était impossible vu la sévérité de sa maladie.

**Figure 1:**
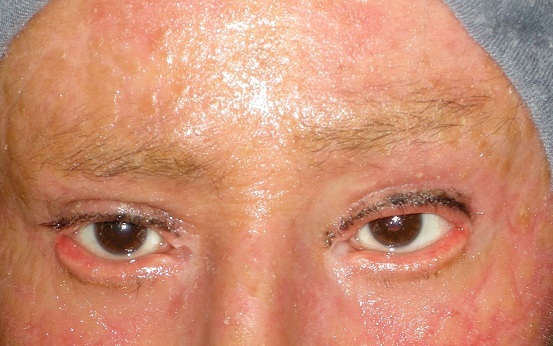
Ectropion bilatéral

